# Review: application of the Safety Attitudes Questionnaire (SAQ) in primary care - a systematic synthesis on validity, descriptive and comparative results, and variance across organisational units

**DOI:** 10.1186/s12875-024-02273-z

**Published:** 2024-01-25

**Authors:** Anne Estrup Olesen, Marie Haase Juhl, Ellen Tveter Deilkås, Solvejg Kristensen

**Affiliations:** 1 Department of Clinical Pharmacology, Aalborg University Hospital, Mølleparkvej 8a, 9000 Aalborg, Denmark; 2 Department of Clinical Medicine, Aalborg University, Selma Lagerløfs Vej 249, 9260 Gistrup, Denmark; 3 Health Services Research Unit, Akershus University Hospital, Sykehusveien 25, Oslo, Norway; 4 Aalborg University Hospital, Psychiatry, Mølleparkvej 10, 9000 Aalborg, Denmark

**Keywords:** Safety attitude questionnaire, Patient safety, Validation, Intervention, Primary care

## Abstract

Patient safety research has focused mostly on the hospital and acute care setting whereas assessments of patient safety climate in primary health care settings are warranted. Valid questionnaires as e.g., the Safety Attitudes Questionnaire (SAQ) may capture staff perceptions of patient safety climate but until now, an overview of the use of SAQ in primary care has not been systematically presented. Thus, the aim of this systematic review is to present an overview of SAQ used in primary care.

**Methods** The electronic databases: PubMed, Embase, Cinahl, PsycInfo and Web of Science were used to find studies that used any version of SAQ in primary care. Studies were excluded if only abstract or poster was available, as the information in abstract and posters was deemed insufficient. Commentaries and nonempirical studies (e.g., study protocols) were excluded. Only English manuscripts were included.

**Results** A total of 43 studies were included and 40 of them fell into four categories: 1) validation analysis, 2) descriptive analysis, 3) variance assessment and 4) intervention evaluation and were included in further analyses. Some studies fell into more than one of the four categories. Seventeen studies aimed to validate different versions of SAQ in a variety of settings and providers. Twenty-five studies from fourteen different countries reported descriptive findings of different versions of SAQ in a variety of settings. Most studies were conducted in primary health care centres, out-of-hours clinics, nursing homes and general practice focusing on greatly varying populations. One study was conducted in home care. Three studies investigated variance of SAQ scores. Only five studies used SAQ to assess the effects of interventions/events. These studies evaluated the effect of electronic medical record implementation, a comprehensive Unit-based Safety Program or COVID-19.

**Conclusion** The synthesis demonstrated that SAQ is valid for use in primary care, but it is important to adapt and validate the questionnaire to the specific setting and participants under investigation. Moreover, differences in SAQ factor scores were related to a variety of descriptive factors, that should be considered in future studies More studies, especially variance and intervention studies, are warranted in primary care.

**Trial registration** This systematic review was not registered in any register.

## Introduction

The extent and seriousness of the healthcare problem related to patient safety culture are significant and have garnered increasing attention in recent years [1]. Patient safety is a health care discipline that emerged with the evolving complexity in health care systems and the resulting rise of patient harm in health care facilities. Patient safety culture refers to the attitudes, perceptions, values, and behaviours within healthcare organizations that impact patient safety. It encompasses how healthcare professionals, administrators, and staff perceive and prioritize patient safety and how this perception translates into their actions.

Patient safety management emphasizes a system of care delivery that prevents and reduces risks, errors and harm that occur to patients during provision of health care [2]. Such a system is built on a culture of safety that involves influence from politicians, organizations, health care planners, healthcare professionals, patients and their relatives [3]. By providing the main point of contact for patients and especially for those with complex care needs, primary health care can make health systems more safe, efficient, effective, and equitable [4]. To avoid risk and harm there is a continuous need to improve patient safety culture in healthcare worldwide requiring assessments of measurable dimensions.

Safety climate is a term that generally refers to the measurable components of ‘‘safety culture’’ such as management behaviours, safety systems, and employee perceptions of safety [5]. Thus, when using questionnaires to study group-level perceptions, the most appropriate term to use is climate (e.g., safety climate, or teamwork climate) [6]. Self-administered questionnaires have been developed as means of measuring quantitively important aspects of safety climate. Several surveys to measure staff perceptions of patient safety climate in hospital settings exist [7, 8].

The Safety Attitudes Questionnaire (SAQ) is an example of a widely used survey and was originally designed in 2006 as a staff survey that measures 6 safety-related climate domains in 60 items. The SAQ elicits a snapshot of the safety culture through surveys of self-reported health care worker perceptions [6]. The questionnaire was invented in Texas [6] and has been validated and frequently used internationally. Later modified versions have been developed for intensive care units (SAQ-ICU) [9], and for the outpatient setting, the SAQ-Ambulatory Version (SAQ-A) has been developed and validated [10, 11]. A short form generic version (SAQ-SF) [12], including 31 scaled items equivalent to 6 dimensions was developed and is now recommended for use [13]. Detailed descriptions on SAQ analysis have been published [6, 10, 14–16].

Patient safety research has focused mostly on the hospital setting and acute care setting where less attention has been paid to the primary care [17]. However, as adverse events including medication errors and delayed diagnoses are challenges in primary care, introduction of the concept of and maintaining a safety culture, should have a positive impact on safety outcomes [2]. Safety culture can be assumed to vary between primary and secondary health care due to different organizational structure and administrative and clinical processes. It has been suggested that differences may partly be explained by the fact that primary care settings focus more on multidisciplinary teamwork to care for a complex group of patients [18].

The definition of primary healthcare encompasses various healthcare organizations and provider groups and varies across countries [19]. Examples include general practice, ambulatory care, nursing homes, and home care. Differences between private and public primary care systems exist worldwide.

Primary care medication management constitutes a complex health care system [20] and it has been demonstrated that improved safety and teamwork climate as measured by SAQ are associated with decreased patient harm and severity-adjusted mortality [21]. With increasing political and scientifical focus on primary care as a major target of patient safety improvement, assessments of patient safety climate in primary health care settings are warranted. Until now, only few studies have been conducted. Different initiatives have been developed to improve the safety culture in nursing and residential homes, such as leadership walkarounds and team training. However, few instruments are available to evaluate the effectiveness of these initiatives and little is also known about the current safety culture of nursing and residential homes [18].

Psychometrically sound questionnaires that have proven reliable and valid for use in research and or clinical settings can be used to determine how staff perceptions of patient safety culture varies across work sites, groups of informants, and domains etc. [22, 23]. Although the SAQ is recommended for global use, an overview of the use of SAQ in primary care has not yet been systematically presented.

Thus, the aim of this systematic review is to describe and synthesize the available literature on SAQ used in primary care.

## Method

The study was a systematic review. Relevant items from The Preferred Reporting Items for Systematic reviews and Meta-Analyses (PRISMA) statement [24] guided this systematic review.

### Literature search

A literature search was conducted, with assistance from a librarian, to find studies that used any version of SAQ in primary care. The electronic databases: PubMed, Embase, Cinahl PsycInfo and Web of Science was used. The search was carried out by first author with assistance from the Medical Librarian at Aalborg University Hospital. The search terms used were: “Safety attitude* questionnaire*” AND “Primary health care” OR "primary healthcare" OR "primary care" OR "primary health sector" OR "primary sector" OR "first line care" OR "primary medical care" OR "ambulatory setting*" OR out-patient* OR outpatient*. The search was limited to 2006-2023 due to the fact, that the first SAQ publication was published in 2006. The searches were conducted March 14^th^, 2023 and updated November 7^th^ 2023. In addition, snowballing using citations, and references to other publications on SAQ in primary care was used to search for additional studies.

### Inclusion and exclusion criteria

Studies were excluded if only abstract or poster was available, as the information in abstract and posters was deemed insufficient. Commentaries and nonempirical studies (e.g., study protocols, editorials) were excluded. Only English manuscripts were included. Studies referring to primary care were included.

### Synthesis of study results and framework for analysis

A synthesis was carried out focusing on a qualitative analysis of the information obtained for each for the four themes. In an analytical framework, classification was used to charting the data by organising concepts into themes to systematically select relevant outcomes to compare properties of SAQ in primary care.

## Results

### Literature search, selection and classification

**Fig. 1:**
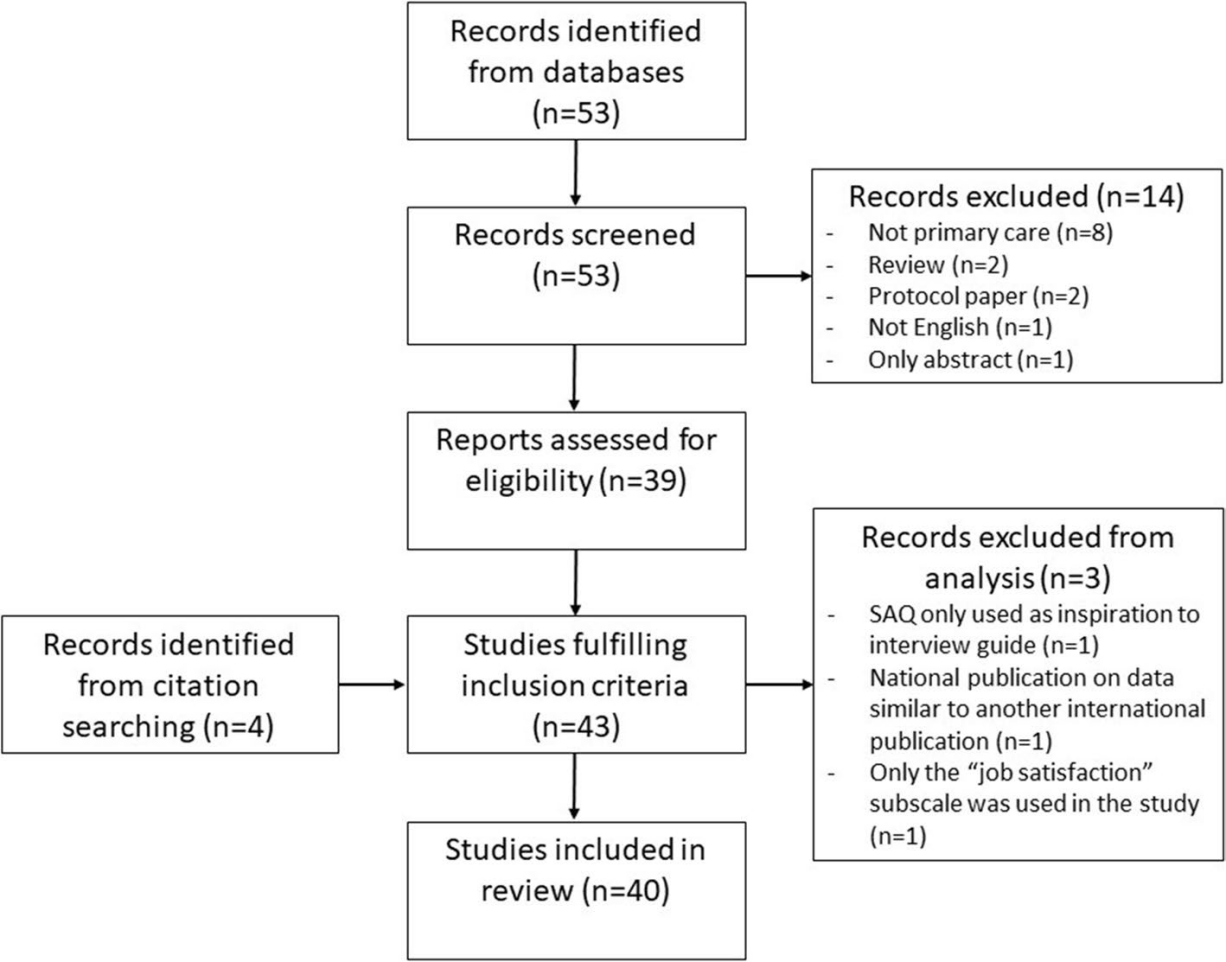
Study selection flowchart

The literature search resulted in 53 publications after deletion of duplicates. The first study was published in 2007. Additional four studies were identified via snowballing. A total of 43 studies were included based on in- and exclusion-criteria. A least two authors screened each record independently to evaluate if the studies met the inclusion criteria. Due to the low number of retrieved studies, no studies were removed before screening and no automation tools were used in the process. Three of the 43 studies were not included in further analyses. The study selection flowchart is illustrated in Fig. 1.

### Framework for analysis and outcomes

**Fig. 2:**
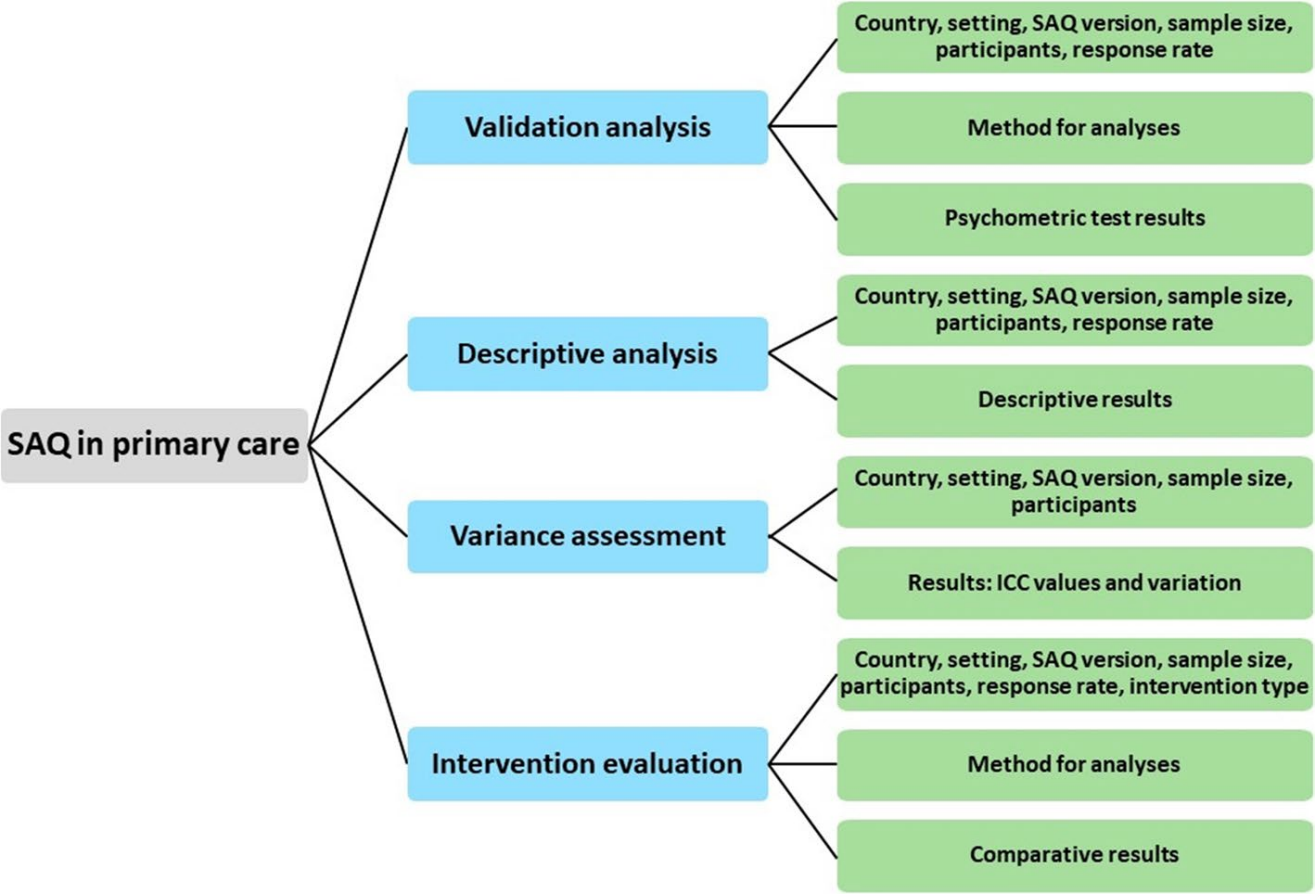
The analytical framework for studies on the use of SAQ in primary care. The classification resulted in four themes (blue boxes). For each theme relevant outcomes (green boxes) were extracted

An analytical framework classification charted the data by organising concepts into themes. Relevant outcomes were systematically selected to compare properties of SAQ in primary care (Fig. 2). All authors contributed to the analytical framework and agreed on the synthesis and the relevant outcomes. One German study validated a newly developed and unique safety climate questionnaire, based partly on SAQ-A, for use in German general practices [25]. In comparison with the SAQ-A, only 17 of 30 items and two of six dimensions (team climate and job satisfaction) remained. Although, the study started out with the SAQ-A, the different interpretation and content of factors showed that a brand new questionnaire was developed (FraSiK) [25]. Thus, this study was not included in further analysis. Results from a Slovenian study were presented both internationally and nationally [17, 26]. We decided only to include the international study, thus the national Slovenian study was not included in further analysis [26]. Another study investigated the practice environment of primary care nurses and used the job satisfaction subscale of SAQ-SF to assess job satisfaction [27]. As only one of six dimensions were used, the study was not included in further analysis.

The 40 remaining studies were divided into four analytic categories: 1) validation analysis, *N*=17, 2) descriptive analysis, *N*=25, 3) variance assessment, *N*=3 and 4) intervention evaluation, *N*=5. Nine studies were included in more than one category.

**Fig. 3:**
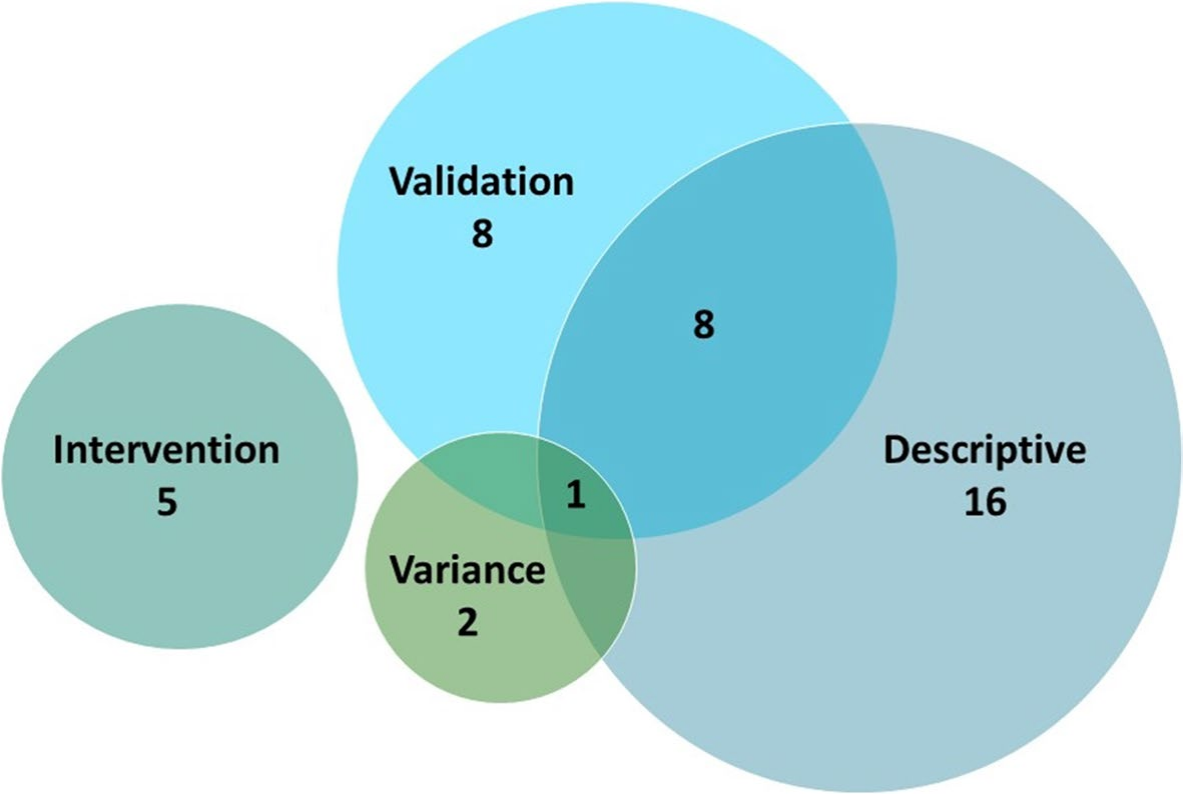
Number of studies included in each of the four analytical themes

The analytical key issues and themes were used to synthesize the findings and present results. Figure 3 provides an overview of number of studies included in the four analytic themes.

### Validation analysis

Seventeen studies from thirteen different countries have validated different versions of SAQ including SAQ-A, SAQ-AV (*n*=11), SAQ-SF (*n*=4), a modified Chinese version (CSAQ, *n*=1) and a non-specified SAQ version (*n*=1) in a variety of settings and participants in primary care [10, 17, 18, 28–41] (Table 1). Doctors and or nurses participated in all studies except for one carried out in pharmacies. Sample sizes varied from 211 to 7427 invitees and response rates varied between 8.7% and 99.4% (Table 1). SAQ-AV are derived from the SAQ-A versions and in some studies, SAQ-AV and SAQ-A were used interchangeably. Psychometric properties of the questionnaires in each of the studies are presented in Table 1. Reliability was mostly assessed using measures of internal consistency including Cronbach’s alpha, but acceptable threshold levels varied slightly across studies (0.60-0.70). Seven studies [10, 17, 32, 33, 35, 38, 41], had response rates above the anticipated 60% [15] with the number of completed questionnaires varying from 154 to 4090 These studies were carried out in a variety of settings. All seven studies reported Cronbach’s alphas above 0,6 and different factor structures (between 4 and 6 factors). Factor analyses, both exploratory and confirmatory, were used to validate the constructs of the SAQ questionnaires (Table 1), leading to identification and acceptance of different factor structures across studies, most often a six-factor structure was confirmed.

**Table 1:** Validation analysis studies of SAQ

**First author (year)**	**1. Country** **2. Setting** **3. Participants**	**SAQ version**	**1. Sample size** **2. Completed questionnaires** **3. Response rate**	**1. Psychometric test results (Cutoff values)** **2. Reliability** **3. EFA** **4. CFA**	**1. Analyses** **2. Factor structure** **3. Confirmed (Yes/No)**
Gabrani (2016) [35]	1. Albania 2. Primary health care centers 3. Specialist physicians, general physicians and nurses	SAQ-A	1. NA 2. 526 3. 99.4%	1. Cronbach’s α=0.62-0.82 (0.7) 2. NA 3. SRMR=0.078, RMSEA=0.049 (0.10), CFI=0.98 (0.90)	1. CFA 2. 6 factors 3. Yes
Mesaric (2020) [36]	1. Croatia 2. Out-of-hours primary care service 3. Medical doctors and support medical staff (including registered nurses and administrative staff)	SAQ-AV	1. 358 2. 185 3. 51.7%	1. Cronbach’s α=0.79-0.93, Cronbach’s α-total=0.95 (0.7) 2. Kaiser-Meyer-Olkin measure=0.82 (0.5), McDonalds’ ω=0.13-0.56, CITC=0.11-0.72 (0.3) 3. NA	1. EFA 2. 6 factors 3. NA
Hussein (2022) [37]	1. Egypt 2. Primary health care units and general Hospital (tertiary level of care) 3. Physicians, dentists, pharmacists, nurses and technicians	CSAQ	1. 240 2. NA 3. NA	1. Cronbach’s α-total=0.915 (NA) 2. NA 3. NA	1. NA 2. 7 factors 3. NA
Demurtas (2020) [38]	1. Italy 2. Out-of-hours service 3. Out-of-hours doctors	SAQ-AV	1. 692 2. 491 3. 71%	1. Cronbach’s α=0.710-0.917 (NA) 2. Kaiser-Meyer-Olkin measure=0.843 3. CFI=0.815 (close to 1), TLI=0.799 (close to 1), RMSEA=0.077 (0.10)	1. EFA and CFA 2. 4 factors 3. Yes
Khamaiseh (2020) [39]	1. Jordan 2. Primary health care centers 3. Registered nurses, assistant nurses and associated nurses	SAQ-SF	1. NA 2. 644 3. NA	1. Cronbach’s α-total=0.90 (NA) 2. NA 3.NA	1. NA 2. 6 factors 3. NA
Bondevik (2014) [11]	1. Norway 2. Clinics: Out-of-hours casualty clinics and regular general practices 3. Nurses, medical doctors and “unknown”	1. SAQ-AV	1. 510 2. 266 3. 52%	1. Cronbach’s α=0.67-0.83, Cronbach’s α-total=0.886 (0.70) 2. NA 3. CFI=0.86, P-value <0.001, RMSEA=0.07, χ^2^/df=1.82	1. CFA. 2. 5 factors 3. Yes
Bondevik (2019) [41]	1. Norway 2. Nursing homes 3. Registered nurses, nursing assistants, health workers, kitchen personnel, laundry personnel, secretary and other personnel	SAQ-A	1. 463 2. 288 3. 62.2%	1. Cronbach’s α=0.655-0.786 (good if between 0.70 and 0.90, and acceptable if above 0.60) 2. NA 3. CFI=0.891 (0.90), P-value<0.001, χ^2^/df=1.846 (0.05), RMSEA=0.054 (0.08), Pclose=0.144 (>0.05), Hoelter 0.05=176 (200)	1. CFA 2. 6 factors 3. Yes
Ogaji (2021) [29]	1. Nigeria 2. Primary and tertiary level of care: The Federal Medical Center and selected health centers 3. Doctors, nurses, laboratory staff, pharmacy staff, community health practitioners and support staff	SAQ-AV	1. 812 2. 436 3. 53.7%	1. Cronbach’s α=0.62-0.76, Cronbach’s α-total=0.91 (0.70) 2. NA 3. NA	1. NA 2. 8 factors 3. NA
Ferreira (2022) [34]	1. Portugal 2. Primary health care units 3. Physicians, doctors in pre-career training, nurses and technical assistants	SAQ-SF	1. 7427 2. 649 3. 8.7%	1. Cronbach’s α=0.069-0.788, Cronbach’s α(tot)=0.86 (0.70) 2. Na 3. NA	1. NA 2. 6 factors 3. NA
AlMaani (2021) [30]	1. Saudi Arabia 2. Primary health-care centers distributed among one region in three sectors 3. Physicians, nurses, pharmacists, and allied health personnel employees	SAQ	1. 344 2. 288 3. NA	1. Cronbach’s α=0.73-0.85, Cronbach’s α-total=0.86 (0.70) 2. NA 3. NA	1. NA 2. 6 factors 3. NA
Klemenc-Ketis (2017) [31]	1. Slovenia 2. Out-of-hours primary care clinics 3. Physicians, graduate nurses, nurse managers, trainees, nurses, radiology technicians and office managers	SAQ-AV	1. 438 2. 250 3. 57.1%	1. Cronbach’s α=0.587-0.791, Cronbach’s α-total=0.922 (NA) 2. Kaiser-Meyer-Olkin measure = 0.897 (NA), Bartlett test<0.001 (NA) 3. NA	1. EFA 2. 5 factors 3. NA
Klemenc-Ketis (2018) [17]	1. Slovenia 2. Community health centers covering one municipality 3. All employees with a leadership role (e.g., physicians, dentists, registered nurses, nurse assistants, administrative staff etc.)	SAQ-SF	1. 211 2. 154 3. 73.0%	1. Cronbach’s α=0.781-0.874, Cronbach’s α-total=0.963 (0.7=acceptable, 0.8=good and 0.9=excellent) 2. Kaiser-Meyer-Olkin measure=0.824 (0.8), Bartlett test<0.001 (0.001) 3. P value < 0.001 relative chi-square= 1.636, CFI = 0.874 (0.90-1.00), NFI = 0.737 (0.90), RMSEA= 0.064 (0.05)	1. EFA and CFA 2. 6 factors 3. No
Nordén-Hägg (2010) [32]	1. Sweden 2. Pharmacies 3. Pharmacists, prescriptionists, pharmacy technicians, pharmacy assistants and "Others"	SAQ-SF	1. 6683 2. 4090 3. 61.2%	1. Cronbach’s α=0.72-0.89 (NA) 2. NA 3. CFI=0.886-0.903 (0.90), RMSEA=0.050-0.060 (0.08)	1. CFA 2. 6 factors 3. Yes
Buljac-Samardzic (2016) [18]	1. The Netherlands 2. Nursing and residential homes 3. Employees who provide direct care to clients. Teams of nurse’s aides, registered nurses and a geriatric specialist (doctor). Occupational, speech and physical therapist and licensed practical nurses	SAQ-AV	1. NA 2. 521 3. 53%	1. Cronbach’s α=0.56-0.80 (0.70/0.50) 2. Kaiser-Meyer-Olkin measure=NA (0.60), Bartlett’s test=NA (0.40) 3. NA	1. EFA 2. 6 factors 3. NA
Smits (2017) [28]	1. The Netherlands 2. Out-of-hours general practitioner cooperatives and call centers 3. General practitioners, triage nurses and other personnel	SAQ-AV	1. 1974 2. 853 3. 43.2%	1. Cronbach’s α=0.49-0.86 (0.70) 2. Kaiser-Meyer-Olkin measure=0.90 (0.5), Bartlett’s test: χ^2^=478.3; df= 351; p < .001. 3. Details not reported	1. EFA and CFA 2. 5 factors 3. No
Singh (2008) [33]	1. USA 2. Primary care offices 3. Physicians, nursing staff, admin staff, unknown position	SAQ-A	1. 252 2. 160 3. 63%	1. Cronbach’s α=0.58-0.77 (0.70) 2. NA 3. NA	1. NA 2. 6 factors 3. NA
Modak (2007) [10]	1. USA 2. Academic, urban, outpatient practices 3. Physicians, nurses, managers, medical assistants	SAQ-A	1. 282 2. 2513. 69%	1. Cronbach’s α=0.68-0.86 (NA) 2. NA 3. CFI=0.973 (0.90), TLI=0.977 (0.90), RMSEA=0.067 (0.08)	1. CFA 2. 6 factors 3. Yes

In summary different versions of SAQ have been validated for different settings. However, heterogeneity was high in how extensively the different SAQ adaptations were validated.

### Descriptive analysis

Twenty-five studies reported descriptive results of SAQ in primary care [10, 18, 29, 30, 33, 34, 37–40, 42–56] (Table 2). The studies came from fourteen different countries. Most studies were conducted in primary health care centres, out-of-hours clinics, nursing homes and general practice. Only one study from Brazil was conducted in home care [44]. The studies used different versions of SAQ including SAQ-SF, SAQ-AV, and a modified Chinese version (CSAQ). Sample sizes of invitees ranged from 140-1974 (Table 2). A variety of participants were included in the studies and response rates ranged from 8.7%-97.8%. The Job satisfaction factor obtained highest scores in some studies [39, 44, 45, 51] and lowest scores in one study from Slovenia [50]. Perception of Management received lowest scores in some studies [37, 39, 44]. Results from the twenty-five studies are presented in Table 2.

**Table 2:** Descriptive analysis studies using SAQ

**First author (year)**	**1. Country** **2. Setting** **3. Participants**	**SAQ version**	**1. Sample size** **2. Completed questionnaires** **3. Response rate**	**Results – short summary**
Paese (2013) [42]	1. Brazil 2. Primary health centres 3. Community health agents, nursing technicians, nurses	SAQ	1. NA 2. 96 3. NA	No difference between the three professional categories regarding perceived attitudes toward safety when analysed in a general context. Working conditions, patient safety culture, communication and management of the healthcare centre safety attitudes were perceived differently by the community health agents compared to nursing technicians and nurses.
Mazzuco de Souza (2019) [43]	1. Brazil 2. Primary health care 3. Nurses, nursing technicians, community health agent, doctors, dentists, oral health assistants, nursing auxiliaries, physiotherapists, physical educators, doctors, psychologists, pharmacists, nutritionists, social workers, speech therapists	SAQ-AV	1. 342 2. 254 3. 74.3%	No associations were found between positive culture and gender, age, degree of education or professional group. Positive culture was related to sector of performance and having five to 12 years of work.
Lousada (2020) [44]	1. Brazil 2. Primary health care centres and home care settings 3. Community health agents, nursing technicians, physicians, nurses, physiotherapists, administrative supporters, psychologists, social workers, speech therapist, other	SAQ	1. 164 2. 147 3. 86.1-86.6%	Job satisfaction obtained the highest value. Perception of management and working conditions had the lowest scores, and this result was related with long time of experience. Males gave higher scores for safety climate, perception of stress, management perception and total SAQ than women. Home care professionals gave higher scores than primary care professionals for all domains, except perception of stress.
El Shafei (2019) [45]	1. Egypt 2. Primary health care facilities 3. Physicians, nurses, pharmacists, managers	SAQ-AV	1. 204 2. 130 3. 63.7%	Participants belonging to age group older than or equal to 50 scored higher in both job satisfaction and working conditions. Managers showed the highest response rate (100%).
Hussein (2022) [37]	1. Egypt 2. Primary health care units and general hospital (tertiary level). 3. Physicians, dentists, pharmacists, nurses, technicians	CSAQ	1. NA 2. 240 (120/120) 3. NA	The total mean score of patient safety attitude was higher among those aged ≥ 40 years, male respondents, married, MD educated, nurses and those who had patient safety training. Tertiary health care workers had higher mean scores of teamwork climate, perception of management, job satisfaction, working conditions, and stress recognition’ and the overall CSAQ score.
Demurtas (2020) [38]	1. Italy 2. Out-of-hours service setting 3. Physicians	SAQ-AV	1. 692 2. 491 3. 71%	Males scores were higher than females scores for communication, safety climate, Perception of management and burnout risk. Providers in the 31-40 age group had lower factor mean score for communication, safety climate and perception of management than younger and older providers. Providers with more years of working experience had higher mean score for communication and safety climate than those with less experience. Providers with more than 20 years of work in the same clinic had higher mean score of perception of management than providers working fewer years.
Khamaiseh (2020) [39]	1. Jordan 2. Primary health-care centres 3. Nurses	SAQ-SF	1. NA 2. 644 3. NA	No significant difference in the perception of patient safety was found between genders or age groups. Educational level was associated to safety climate and perception of management and job position was associated to perceptions of management.
Alameddine (2015) [46]	1. Lebanon 2. Primary health-care centres 3. Physicians, dentists, nurses, technicians, nutritionists, pharmacists, social workers, midwives	SAQ-A	1. NA 2. 943 3. 44%	The highest response rate was from nurses (82 %) followed by specialists (43 %). Dentists, general practitioners, and allied health professionals had response rates of 34-36%. Providers with the highest SAQ score had higher odds to report a higher readiness on the appropriateness, efficacy, management, and personal valence Readiness for Organization Change subscales
Samsuri (2015) [47]	1. Malaysia 2. Public hospitals and health clinics 3. Pharmacists	SAQ (Pharmacy version)	1. 140 2. 117 3. 83.6%	Apart from stress recognition, those who worked in health clinics scored higher than those in hospitals. Higher scores (overall score as well as score for each domain except for stress recognition) were associated with fewer numbers of medication errors reported. In contrast stress recognition was associated with increased number of medication errors reported.
Ogaji (2021) [29]	1. Nigeria 2. The Federal Medical Centre and health centres (primary and tertiary) 3. Doctors, nurses, laboratory staff, pharmacy staff, community health practitioners, support staff	SAQ-AV	1. 812 2. 436 3. 53.7%	76.5% from the primary health care facilities and 40.2% from the tertiary responded to the questionnaire. Scores were significantly higher in primary health care facilities compared to tertiary health care facilities except for job satisfaction.
Bondevik (2014) [40]	1. Norway 2. Out-of-hours casualty clinics and general practices 3. Doctors, nurses (incl. registered nurses, medical secretaries, and bioengineers)	SAQ-AV	1. 510 2. 266 3. 52%	72% of the nurses and 39% of the doctors answered the questionnaire. Health care providers in general practitioner practices had significant higher mean scores on the factors safety climate and working conditions, compared with those working in the out-of-hours clinics. In general practitioner practices, male health professionals scored significantly higher than female on teamwork climate, safety climate, perceptions of management and working conditions. Older health care providers scored significantly higher than younger on safety climate and working conditions. In the out-of-hours clinics, nurses scored significantly higher than doctors on Safety climate and Job satisfaction.
Bondevik (2017) [48]	1. Norway 2. Nursing homes 3. Registered nurses, nursing assistants, health workers, kitchen personnel, other personnel	SAQ-AV	1. 463 2. 288 3. 62.2%	Response rates varied between 56.9% and 72.2% across the five nursing homes. Increasing age and higher job position among the healthcare providers were associated with significantly increased mean scores for the patient safety factors teamwork climate, safety climate, job satisfaction and working conditions. Not being a Norwegian native speaker was associated with a significantly higher mean score for Job satisfaction and a significantly lower mean score for stress recognition. Neither professional background nor work experience were significantly associated with mean scores for any patient safety factor.
Ferreira (2022) [34]	1. Portugal 2. Primary health care units 3. Physicians, doctors in pre-career training, nurses and technical assistants working	SAQ-SF	1. 7427 2. 676 3. 9.1%	The lowest scores in team environment were obtained for the categories of nurse, technical assistant, and customized healthcare units. The lowest median score in the safety climate domain was obtained in the customized healthcare units The lowest scores in the Job satisfaction domain were obtained among male respondents and in the customized healthcare units. The lowest median scores in management perception were obtained among male respondents and in the customized healthcare units. In the stress recognition domain, as the age of the respondent increased, the obtained SAQ-SF median score decreased, and as the length of service at the respondent’s current workplace increased, so did the obtained score. The total SAQ-SF median scores were higher among female respondents, in one workplace and in two types of primary care units.
AlMaani (2021) [30]	1. Saudi Arabia 2. Primary health-care centres 3. Nurses, technologists, physicians, pharmacists, others	SAQ	1. NA 2. 288 3. NA	The score of teamwork and stress recognition was higher among females. Whereas perception of management was higher among males. All factors and the overall score were higher in providers less than 40 years compared to older providers. Perception of management was lower among physicians. The overall score for safety attitudes was higher among those with less than 10 years' experience. The overall safety culture score was significantly higher among managers.
Elsayed (2020) [55]	1. Saudi Arabia 2. Primary health-care centres 3. Nurses	SAQ	1. NA 2. NA 3. 314	A difference between nurses’ attitude and gender was found, also there was a difference between nurses’ attitude and years of experience. No difference between nurses’ attitude and their age, educational qualifications, and staff position.
Klemenc-Ketis (2017) [49]	1. Slovenia 2. Out-of-hours-health care clinics 3. Physicians, nurse practitioners, nurse managers, trainees, practice nurses, radiology technicians, office managers	SAQ-AV	1. 438 2. 250 3. 57.1%	Differences were found across different Slovenian regions in perception of management, job satisfaction, communication, and the overall total SAQ-AV score. Physicians, practice nurses, those working in variable shifts and those working full-time had significantly higher total SAQ-AV scores when compared to the other categories.
Klemenc-Ketis (2017) [50]	1. Slovenia 2. Out-of-hours health care clinics 3. Physicians, nurse practitioners (nurses with a bachelor’s degree), practice nurses	SAQ-AV	1. 438 2. 250 3. 57.1%	Overall perceived safety culture was not different between professional groups. Perceptions of management was scored significantly lower by nurse practitioners than by physicians and practice nurses, whereas physicians scored safety climate significantly lower than practice nurses and nurse practitioners
Zúñiga(2015) [56]	1. Switzerland 2. Nursing homes 3. Care workers of all educational levels (e.g., registered nurses (25%), licensed practical nurses, nurse aides) if they worked in direct care of the nursing home residents.	SAQ	1. 4307 (from 402 care units and 74 additional teams in 156 nursing home facilities) 2. NA 3. 78%	A combined factor of Teamwork and Resident Safety Climate with a total of 10 items was used. The facility response rate ranged from 40% to 100%. Higher teamwork and safety climate were only related to lower rationing in the subscales activities of daily living and caring, rehabilitation, and monitoring. In contrast, better teamwork and safety climate was related to higher rationing in social care.
Buljac-Samardzic et al. 2015 [18]	1. The Netherlands 2. Nursing and residential homes 3. Employees who provide direct care to clients. Licensed nurses, aides, registered nurses	SAQ-AV	1. 983 2. 521 3. 53%	The response rate per organisation varied from 40.2% to 81.4% Overall, the scores from the nursing and residential homes differed significantly from the benchmark settings. The safety climate and working conditions in nursing and residential homes were significantly higher rated than in the inpatient setting, but significantly lower than in the intensive care unit and ambulatory setting. Nursing homes scored significantly higher on teamwork climate, job satisfaction and perception of management in comparison with residential homes.
Smits (2018) [51]	1. The Netherlands 2. Out-of-hours general practitioner cooperatives 3. GPs, triage nurses	SAQ-AV	1. 1974 2. 853 3. 43%	Gender was not associated with any of the patient safety factors. Older healthcare providers scored significantly higher than younger on safety climate and perceptions of management. Triage nurses scored significantly higher than GPs on each of the five patient safety factors. More working experience was positively related to higher team- work climate and communication openness.
Modak** (**2007) [10]	1. USA 2. Academic, urban, outpatient practice 3. Physicians, nurses, manager, medical assistants, support staff	SAQ-A	1. 409 2. 282 3. 69%	Physicians had the least favourable attitudes about perceptions of management while managers had the most favourable attitudes. Nurses had the most positive stress recognition. All providers had similar attitudes toward teamwork climate, safety climate, job satisfaction, and working conditions.
Singh (2008) [33]	1. USA 2. Primary care offices 3. Physician, nursing staff, administrative staff, unknown position	SAQ-A	1. 252 2. 160 3. 63%	Comparing eight practices, differences were found among sites on all subscales except stress recognition. No differences among respondent groups on any subscale were found.
Holden (2009) [52]	1. USA 2. Air Force ambulatory care facilities 3. Physicians, nurse practitioners, physician assistants, registered nurses, pharmacists, technicians	SAQ	1. 328 2. 213 3. 65%	Differences on total safety scores based on age, with staff members younger than 31 years scoring lower on the overall safety score as compared with the 32- to 41-year age group and those 42- to 63-year age group. No significant differences among the professional groups on the total patient safety scores or on 5 of the 6 subscales. Significant difference on the Stress recognition subscale, with technicians scoring less than 4 of the 5 other professional groups.
Holden (2010) [53]	1. USA 2. Military ambulatory care clinics 3. Nurses, nurse practitioners, pharmacists, physicians	SAQ	1. NA 2. 107 3. 65%	No significant difference among professional groups on the total weighted safety score or any of the subscales. There were, however, five specific questions with significant group differences: Pharmacists reported higher support to care for patients, morale, and knowledge of the names of their co-workers. Additionally, they were less likely to recognise the impact of fatigue on routine performance and more likely to report making errors that had potential to harm patients. Nurse practitioners and nurses were comparable to pharmacists, with the former also scoring high on the teamwork question related to name recognition and the latter scoring low in recognizing the impact of fatigue on performance.
Miller (2019) [54]	1. USA 2. Academically affiliated ambulatory care 3. Administrative support staff, clinical support staff, managers, providers	SAQ	1. 828 2. 722 3. 87%	Associations were found between safety reporting rates and SAQ scores for overall culture and four safety culture domains: Teamwork climate, safety climate, working conditions, and perceptions of local management. Thus, for every 1-percentage-point increase in overall culture score, there was a 1.9% increase in monthly safety reports. The stress recognition and perceptions of senior management domains did not show a significant correlation with event reporting

Overall differences in SAQ factor scores were related to demographic characteristics as different scores were found when different settings (regions, clinics, practices, and teams), genders, ages, degrees of education, professional groups, time of professional experiences and job types were compared (Table 2).

Three studies compared primary and tertiary health care facilities and found that SAQ-AV results were significantly different between primary and tertiary health care facilities [29, 37, 47]. Two studies found that providers from primary care scored higher than providers from tertiary care [29, 47]. In contrast, another study from Egypt found that tertiary health care workers had higher mean scores of Teamwork climate, Perception of management, Job satisfaction, Working conditions, and Stress recognition’ and the overall CSAQ score [37]. The response rate was lower in tertiary centre than from the primary level of care [29]. Another study compared primary health care centres and home care settings and found that home care professionals gave higher scores than primary care professionals for all domains, except Perception of stress [44].

Several studies reported that males gave higher SAQ-scores than females [11, 37, 38, 44]. However, some studies did not find these differences [39, 43, 51] and others reported higher scores from females in some factors [34]. For example AlMaani et al. reported that the score of Teamwork and Stress recognition was higher among females, whereas Perception of management was higher among males [30].

In general, older employees gave higher scores than younger [11, 37, 38, 45, 48, 51, 52]. However, some studies did not find this association [39, 43] and one study, in contrast found that all factors and the overall score were higher in providers less than 40 years compared to older providers [30]. Another study from Portugal found that in the stress recognition domain, as the age of the respondent increased, the obtained SAQ-SF median score decreased [34].

Some studies investigated the association of SAQ-scores to other variables. For example, one study investigated if SAQ is an indicator for health care providers readiness for reporting quality and found hat providers with the highest SAQ score had higher odds to report a higher readiness on the appropriateness, efficacy, management and personal valence Readiness for Organization Change subscales [46]. Another study investigated safety attitudes of pharmacists and found that higher scores (overall score as well as score for each domain except for stress recognition) correlated negatively with number of reported medication errors [47]. In contrast, Miller et al. studied the relationship between safety culture and voluntary adverse event reporting in a regional ambulatory care group and found that for every 1-percentage-point increase in overall climate score, there was a 1.9% increase in monthly safety reports [54]. Another study investigated the relationship between safety climate and implicit rationing of nursing care and found that higher safety climate was only related to lower rationing in the subscales activities of daily living and caring, rehabilitation and monitoring whereas better safety climate was related to higher rationing in social care [56].

In summary, differences in SAQ factor scores were related to a variety of factors, that should be considered in future studies.

### Variance assessment

Only three studies investigated variance in SAQ-scores across organizational units at different levels, one study from the Netherlands and two studies from Norway [18, 22, 23] (Table 3).

**Table 3:** Variance assessment studies using SAQ

**First author (year)**	**1. Country** **2. Setting** **3. Participants**	**SAQ version**	**Sample size**	**Results – ICC values**	**Variation**
Deilkås (2019) [23]	1. Norway 2. General practices and out-of-hours clinics 3. Medical doctors, registered nurses, medical secretaries, and bioengineers	SAQ-A	510 primary health care providers were invited. 17 GP practices and 7 Out-of-hours clinics. 266 answered	Teamwork climate	14.4%
				Safety climate	16.4%
				Job satisfaction	7.1%
				Working condition	14.6%
				Perception of management	12.1%
				Stress recognition	NA
Deilkås (2019) [22]	1. Norway 2. Nursing homes 3. Most of invited employees were registered nurses or nursing assistants	SAQ-A	5 nursing homes where 765 employees were nested in 34 wards	Teamwork climate	2.76%
				Safety climate	11.60%
				Job satisfaction	7.61%
				Working condition	12.81%
				Perception of management	14.07%
				Stress recognition	0.00%
Buljac-Samardzic (2016) [18]	1. The Netherlands 2. Nursing and residential homes 3. Nurse’s aides, registered nurses, and a geriatric specialist (doctor)	SAQ-A	521 caregivers representing 53 teams and 9 units	Teamwork climate	Unit level: 6%, Team level: 15%
				Safety climate	Unit level: 8%, Team level: 11%
				Job satisfaction	Unit level: 10%, Team level: 19%
				Working condition	Unit level: 12%, Team level: 20%
				Perception of management	Unit level: 10%, Team level: 21%
				Stress recognition	Unit level: 1%, Team level: 3%

Different settings and participants were investigated, but all three studies used SAQ-AV. Sample size varied between 510 and 765 invitees, and they were nested into different numbers of units, wards, and teams. Response rates varied per organisation.

One study found that team level variance was higher than unit level variance [18]. Another study found that Intraclass correlation coefficients (ICC) for variance at nursing home level was zero or less than one % for all factor scores [22]. At ward level ICCs for the factors were 10.2% or higher for the factors Safety climate, Working conditions and Perceptions of management, 2.4% or lower for Teamwork climate, Job satisfaction, and zero for Stress recognition [22]. Another study found that staff perceptions varied considerably at the work site level: ICCs were 12.3% or higher for all factors except for Job satisfaction–the highest ICC value was for Perceptions of management: 15.5%. Although most of the score variance was at the individual level, there was considerable response clustering at work unit level, for the general practitioner practices and out-of-hours clinics [23].

In summary, variances in SAQ-scores across organizational units were found.

### Intervention evaluation

Only four studies used SAQ to assess changes in SAQ scored over time during interventions in before-after studies and one study used SAQ to examine association between burnout and other factors among health care workers during COVID-19 in primary care settings (Table 4). The five studies came from Singapore, UK, and USA [16, 57–60] (Table 4). Different settings and participants were studied. Three studies used the SAQ, one study used SAQ-A and one study conducted interviews based on a framework adapted from the SAQ (Table 4). Sample size varied from 14 (the qualitative study) to 11286 invitees. Response rates varied from 14.5% to 96.2%.

**Table 4:** Intervention evaluation studies using SAQ

**First author (year)**	**1. Country** **2. Setting** **3. Participants**	**SAQ version**	**1. Sample size** **2. Completed questionnaires** **3. Response rate**	**Intervention/event**	**Method for analysis**	**Results**
Tan (2020) [60]	1. Singapore 2. Public hospitals and primary health care services involved in the care of Covid-19 cases 3. Doctors, nurses, allied health professionals, support staff, administrative and managerial staff	SAQ	1. 11286 2. 3075 3. 27.2%	COVID-19	Crude and adjusted predictors were performed using mixed linear models with institution as a random effect	High SAQ scores were significantly associated with lower scores of Oldenburg Burnout Inventory
Abhiram (2022) [57]	1. Singapore 2. Public hospitals and primary health care services involved in the care of Covid-19 cases 3. Doctors, nurses, allied health professionals, support staff, and administrative staff	SAQ	1. 10.172 (not provided but calculated) 2. 1475 3. 14.5%	COVID-19	Predictors were investigated using generalized linear mixed model with institution as a random effect	Higher proportion of respondents who scored 75% or above for the safety culture score in each domain when comparing mental well-being in 2021 against the previously published cohort in 2020. Achieving a percentage agree in several SAQ domains had a significant negative association with the primary outcomes
Alboksmaty (2021) [58]	1. UK 2. General practice 3. General practitioners	Interviews based on a framework adapted from the SAQ	1. 14 2. NA 3. NA	COVID-19	A directed content analysis approach was adopted to analyse the interview transcripts	The COVID-19 pandemic affected all levels of the health system in the UK, particularly primary care.
McGuire (2012) [16]	1. USA 2. Medical group practice 3. Primary care providers no further information not provided	SAQ-A	1. T1:123; T2:143; T3:181 2. T1:103; T2:122; T3:142 3. T1: 83.7%; T2: 85.3%; T3:78.5%	Electronic medical record implementation	Chi-square test to calculate P-values assuming independent samples from all three years	All patient safety climate factors improved significantly over the period after implementation of electronic medical record, except for stress recognition
Pitts (2017) [59]	1. USA 2. General internal medicine academic practice 3. Physicians, nurse practitioner, medical assistants, medical office coordinators, front-desk staff member	SAQ	1. 26 2. 25 3. 96.2%	Comprehensive Unit-based Safety Program (CUSP)	Information not provided but providers and staff completed the survey three months before CUSP implementation and six months following the kick-off of CUSP	Following CUSP implementation, respondents were more likely to report knowledge of the proper channels for questions about patient safety, feel encouraged to report safety concerns and believe that the work setting made it easy to learn from the errors of others, although these differences did not reach statistical significance

The first interventional study using SAQ in primary care was performed in USA in 2013. The study was conducted to sequentially measure, evaluate, and respond to safety climate and practice safety concerns following electronic medical record implementation in medical group practice (affiliated with an academic medical centre) [16]. It was demonstrated that safety climate improved over the period after implementation of electronic medical record, with statistically significant improvement in all domains except for stress recognition [16].

In 2017 another American study assessed the impact of Comprehensive Unit-based Safety Program (CUSP) on safety climate and teamwork through a before-after comparison of results on the validated SAQ [59]. The study was conducted in general internal medicine, a suburban, academic practice. Twenty-five providers and staff completed the survey three months before CUSP implementation and six months following the kick-off of CUSP. Compared to before, following CUSP implementation, survey respondents were more likely to report knowledge of the proper channels for questions about patient safety, feel encouraged to report safety concerns and believe that the work setting made it easy to learn from the errors of others. However, these differences did not reach statistical significance [59].

Since 2020, three different studies have used SAQ in the evaluation of the impact of COVID-19 on health care workers [57, 58, 60]. One study from Singapore examined burnout and associated factors among health care workers in public hospitals and primary health care services involved in the care of COVID-19 cases [60]. They found that high SAQ scores were significantly associated with lower scores of Oldenburg Burnout Inventory [60]. Another study from Singapore compared health care workers mental well-being in 2021 against the previously published cohort in 2020 [57]. The study included 1475 respondents (response rate 14.5%). For each factor, % positive was a significantly lower in 2021 than in 2020 [57]. However, results for these two studies were not reported for primary and tertiary care separately.

A study from UK investigated GPs' experiences of how UK COVID-19 policies have affected the management and safety of complex elderly patients, who suffer from multimorbidity, at the primary care level [58]. The setting was general practice where fourteen interviews were conducted. The SAQ was used as one of two theoretical frameworks that were the base for drafting a primary interview guide to explore policies’ impact on management and safety. SAQ was not used in full version as the study only included interviews based on themes adapted from five SAQ factors (Work conditions, Safety environment, Perception of management, Teamwork environment and Stress recognition). The study did not make specific conclusions on culture but concluded that the COVID-19 pandemic affected all levels of the health system in the UK, particularly primary care. Based on the GPs’ perspective, changes to clinical practice have offered opportunities to maintain safe healthcare as well as possible drawbacks that should be of concern [58].

In summary, due to lack of available and comparable studies, no firm conclusions can be drawn on the effects of interventions on SAQ-scores.

## Discussion

### Statement of principal findings

In this systematic review of 40 studies investigating the application of the SAQ in primary care we synthesized validity, descriptive and comparative results, and variance across organisational units. Seventeen studies reported on validation of different versions of SAQ in a variety of primary care settings across thirteen different countries, different participants. Number of participants, response rates and validation methods including set threshold values for accepting the validation results, greatly varied across studies. Twenty-five descriptive studies demonstrated differences in SAQ dimensional scores found between settings (regions, clinics, practices, and teams), genders, ages, degrees of education, professional groups, time of professional experiences and job types. Three studies investigating variance in SAQ scores across different organizational levels found significant and substantial variance at work group unit level. Lastly, five before-after interventional studies used SAQ in the evaluation of an intervention or event such as introducing the electronic medical record, comprehensive Unit-based Safety Program, or the COVID pandemic. Study results indicate that SAQ can be used for detection of changes in patient safety culture over time or to point at associations between outcome measures.

### Strength and weaknesses of the study

Strengths of this review include the systematic literature search and systematic methods of study selection and data extraction. To our knowledge, this is the first review of its kind. It adds to knowledge base of using SAQ as a research and quality improvement instrument to assess patient safety culture, and it structures the knowledge into the four themes studied. Other reviews on patient safety instruments have been published, but they cover a variety of assessment instruments [61, 62]. Also, this study covers primary care. Until now most studies on patient safety climate have been conducted in hospital settings [61–64]. Thus, as expected, we found a very limited number of studies on patient safety culture in primary care settings. Nevertheless, this study synthesises valuable information on patient safety culture, which can be used as a step stone for future studies and application in primary care settings.

The study has some weaknesses. Foremost, this systematic review focused only on articles written in English. Another limitation is that the general definition of primary care is very broad and comprises a variety of different types of healthcare organization including different groups of professional health care providers with highly different tasks, which they pertain to while reporting on perceived patient safety culture. We could have excluded specific primary care settings to create a more uniform group for presentation, but doing so would result in the exclusion of pertinent studies. Our objective was to offer a comprehensive overview of the application of SAQ in primary care practices varying significantly across countries [65], and it would be inappropriate to determine which specific primary care setting is of paramount importance. Patient safety research often involves diverse healthcare settings, patient populations, and methodologies. Including all studies helps account for this variability, enabling a more comprehensive assessment of how patient safety attitudes differ across contexts and patient groups. . Moreover, studies also varied in the methodology and sampling strategies used, which makes synthesis of results across a limited number of studies difficult - as is the case in each of our four themes. However, we accounted for this fact by applying a descriptive synthesis carefully reporting on single studies, as opposed to applying quantitative rating review methodology.

This study did not account for language and cultural disparities predominant in the specific countries in which the reported studies were conducted. Such disparities could possibly introduce bias of a complex and unstudied art, which we cannot know the implications of.

Climate is an emergent property, characterizing groups of individuals. Operationally it is assessed by aggregating individual perceptions to the required unit of analysis. And using the mean to represent the climate for that entity. However, this requires within-unit homogeneity or consensus of perceptions. Without sufficient homogeneity, an aggregate score is not a valid indicator of climate [66]. Thus, not only response rate but also intra-unit variation will affect the results and interpretation. Response rates varied between studies, and not all studies reported precise response rates. Because it has been suggested that response rates below 60%, represent opinions rather than culture and climate, we have assessed results from such studies with caution and not given them weight in the synthesis to minimize the introduction of bias of an unknown kind [15]. We found that some professional backgrounds show higher response rates than others. For example one can speculate if the response rate among medical doctors in general practices is higher than for out-of-hours medical doctor, due to a motivational factor of being an owner/leader and being interested in contributing to the evaluation of their work environment [23]. Additionally, we detected response rate markedly lower for physicians than for non-physicians [67] and the overall response rate was almost twice as high among nurses compared to medical doctors in one study [11]. Moreover, the response rate may vary across units (from 44% to 100%) [67] and settings.

A high variation in response rates across studies was found. It has been discussed that the large number of nursing home employees working part-time may have a higher degree of uncertainty about patient safety [41]. This could possibly affect the willingness to participate in studies on patient safety [41] or introduce information bias. In contrast it could also be speculated that employees working part-time may have more personal resources and are less prone to stress and therefore may be more willing to participate in such studies. It has also been discussed that response rate is highly dependent on the method used for distribution of questionnaires. For example, it was demonstrated that the response rate was much higher for questionnaires distributed in meetings (96%) than for those distributed through the mailing system (50%) [67]. Thus, in summary, big variations in response rates indicate that it may be difficult to achieve response rates above 60% in all settings. Yet, it is highly important to investigate all settings in primary care, and account for low response rates when assessing study validity and reporting results and conclusions.

Synthesised, we found that direct comparison between the included studies in each of our four themes is challenging and should be done with caution. This is also the reason why it was not possible to conduct a systematic review or present quantitative analysis.

### Validation analysis

Most studies confirmed validation of different versions of SAQ for use in primary care. However, it should be considered that of the 17 validity studies, seven studies had response rates above the anticipated 60% [15] with the number of completed questionnaires varying from 154 to 4090 and they were carried out in a variety of settings. In six of the studies nurses and or doctors participated, whereas only pharmacists took part in the 7^th^ study. None of the seven studies investigated other health care worker groups of lower-level education that are often represented in primary care, e.g. social and health care assistants, nursing assistant etc. Heterogeneity was high in how extensively the different SAQ adaptations had been validated, thus it was not possible to use a single rating scale for all studies. As an alternative, we found it relevant to show how well the tools had been validated related to reliability, internal consistency, and construct validity (presented in Table 1). All seven studies reported Cronbach’s alpha above 0.6, and different four to six factor structures were accepted following exploratory factor analysis for these seven studies. Five of the studies confirmed factors by confirmatory analysis.

Each clinical area possesses a unique social fabric of culture, potentially leading respondents who work within the same clinical area to respond more similarly than respondents who are members of different clinical areas [6]. Thus, the structural differences in design of the questionnaire versions may reflect variation in the organization of primary health care systems [65], or it may mean that item wordings trigger different connotations in different languages or with different clinical tasks/responsibilities. For these reasons a wise option might be to compare countries’ SAQ results at the item level rather than at the dimensional level [17]. Moreover, given differences in healthcare systems and culture between countries, factors loading differently is not necessarily a problem and may simply reflect the local context of healthcare and the associated variation [68].

It was discussed that the SAQ-AV should be adapted for support staff that have direct patient contact as 25% of the items did not apply to them and that the SAQ-AV needs to be tested in other outpatient settings [10]. One study evaluated the psychometric properties of the SAQ-SF in employees with a leadership role in community health centres [17]. However, leaders and managers tend to rate safety culture higher than frontline workers [69]. Thus, results from studies based exclusively on leaders’ attitudes should be interpreted with caution as they will not provide precise measures of safety climate in each setting. This will inhibit the actions needed in terms of quality improvement strategies.

Each study adapted the SAQ to the setting investigated. The risk of missing items may increase with increased length of the questionnaire which was also discussed in one study arguing that the length of the 62-item SAQ-AV may have dissuaded participants from completing and returning the questionnaire. It would be desired to have a shorter version of the measure for easy administration so long as the shortened version is valid and reliable” [29].

In some studies, stress recognition was not confirmed as a factor [18, 40]. Similar findings were found in the hospital setting where it has been argued, that it should not be included in the overall safety attitude construct, which the SAQ intends to reflect [70]. It is stated that stress recognition is a dissonant sub-scale of the safety climate construct and that the other subscales refer to the perspectives of respondents on their work areas or broader organisational units; stress recognition is about individual perspectives on abilities [70]. This was supported by e.g. Almaani et al. who also found that stress recognition had the lowest mean indicating that the acceptance of how work is affected by stressors is least recognized among all the sub-scales [30].

One study suggested that Communication and Psychological safety were perceived important safety climate factors since they emerged as individual factors without the study seeking to map them explicitly [31]. Thus, it was suggested that Communication and Psychological safety could be considered possible independent factors in future safety climate surveys [31].

### Descriptive analysis

The 25 descriptive studies demonstrated that differences in SAQ dimensional scores were found between settings (regions, clinics, practices, and teams), genders, ages, degrees of education, professional groups, time of professional experiences, job types and rationing of nursing care. However, direct comparison between studies is complex as the studies came from twelve different countries with different clinical cultures and different health care organization/settings. To that end, it has been discussed that the structure differences of the SAQ versions (used in different countries) may reflect cross-national variation in the nature and structure of primary care, or mean that item wordings trigger different connotations in the different languages [28].

As safety climate scores are likely dependent on educational level, they may, be less comparable among healthcare settings that differ in average educational level [18]. Additionally, clear differences in how healthcare professionals conceptualise ‘patient safety and quality have been demonstrated [71]. Understanding this variance may enable more effective targeting of interprofessional improvement strategies [71]. Reporting errors and safety awareness in hospital setting, gender and demographics, work experience, and staffing levels have also been identified as essential factors [61]. It could also be hypothesised that the likely dependency between safety climate scores and educational level is introduced in the curriculum of different professions’ education or in the political, legal, or structural ties and demands of patient safety at national level [72].

Significant variations in patient safety attitudes are related to age [45]. Higher age is directly related to longer life experience and for most people also working experience which may influence patient safety attitudes. Holden et al. discussed that age differences crossed professional lines and may explain why there can be differences among age groups and no substantial differences among professional groups [52]. Thus, Holden concluded that in terms of policy development, those in leadership positions who are concerned with enhancing team-work may be well served to develop strategies and interventions that target the younger professional staff [52]. This was supported by our study as several studies found that younger providers gave lower SAQ-scores.

Only one study was conducted in home care, although the home care setting is highly relevant regarding patient safety culture [44]. A great patient safety challenge in primary care is medication safety. The situation of drug-related problems in the home care setting has not been well- characterized [73]. A systematic review found that home care patients were predominantly elderly. Not surprisingly, polypharmacy was common in patients ≥ 65 years of age and it was found that patients received 5–14 medications per day [73]. Thus, the home care setting is highly relevant regarding patient safety culture and more studies are warranted.

### Variance assessment

Safety climates vary between work sites in hospitals, and predict where to find risk related to tasks, work environment, staff behaviour, and patient results [23]. This may provide opportunity to direct leadership efforts to where improvement is most needed. One purpose with SAQ is to elucidate variation between organizational units, to be able to use the information to direct improvement efforts to the units with highest need. Thus, it is important to study variance between organizational units in the settings to which the SAQ is adapted [74, 75]. In this study we use data that explicitly reveal variance according to organizational level in primary care to explore if safety culture measurements there provide an equal opportunity. In one of our reviewed studies ICC for variance at nursing home level was zero or less than one percent for all factor scores [22]. The lack of variance across nursing homes was supported by another study that found more variance between work units than between institutions [15]. This means that safety culture measurements also in primary care may provide opportunity to direct leadership efforts to work units where improvement is most needed. It is therefore critical to assess safety culture across all work units in primary care institutions, to uncover worksites that may be more promising candidates for patient safety improvement interventions than others.

### Intervention evaluation

We found only five studies using SAQ to assess the effects of interventions in before-after studies in primary care settings. Only one of the studies used a version of SAQ, that has been validated for use in primary care (SAQ-A) [16] and this was the only study finding that safety climate improved over the period after implementation of electronic medical record, with statistically significant improvement in all domains except for stress recognition [16]. The other studies did either not reach statistical significance, did not report results for primary and tertiary care separately or only used SAQ as a framework for qualitative interviews [57–59]. Moreover, none of the interventional studies were conducted in nursing and residential homes or home care. Thus, no firm conclusions can be drawn from this review.

It has been suggested that interventions that aim to improve one aspect of the safety climate are likely to positively influence other aspects too [18]. Nursing and residential homes should, therefore, not feel obliged to invest in extensive programmes that focus on all safety dimensions at the same time [18]. Additionally it has been suggested that patient safety improvement work in general practices and out-of-hours clinics should not only address all work sites in the same way, but focus on site specific challenges at places with lower scores on specific patient safety climate factors [23]. It has been suggested that differences between safety specialists' and workforce groups' beliefs about how to improve patient safety may impede the successful implementation of patient-safety programmes [76]. Now that SAQ has been validated for use in various settings in primary care it is time to investigate and evaluate interventions that may improve patient safety culture.

### Practical implications for the use of SAQ in primary care

Our analysis found that SAQ has been validated and recommend for use in several countries, descriptive and comparative data have been published, and SAQ has been successfully applied to measure differences in score over time, association to other measures as well as variance across settings in primary care. Thus, SAQ could be implemented in primary care settings to a higher degree than it has been until now.

Our study showed that by only considering variance by ICC at institutional level in primary care, potential for safety culture improvement at work unit level may be masked. An alternative strategy to benchmark institutions is to assess them according to the variance between their subordinate work units. That may be done by assessing institutions by the percentage of work units where 60% or more staff respond positively (with scale score >=75). At this level the safety climate is commonly referred to as “mature”. At the level where 80% of staff in a work unit respond positively (with scale score >=75) the safety climate is commonly referred to as “good”. This terminology is currently used in assessing variance in safety culture between Norwegian hospitals [77]. Such a measure could also be implemented in primary care. It could motivate leaders of institutions and municipalities to engage in direct dialogue with subordinate work units to increase mutual understanding of how staff struggle with their safety culture, and how to improve it.

### Unanswered questions and future research

Variance in SAQ-scores across organizational units were demonstrated. However, understanding the sources of variation in safety culture is still a glaring gap in our knowledge of cultural assessment and a key to deeper understanding and practical application [15]. Thus, more studies on variance in safety climates and possible explanatory factors are warranted.

Although multiple studies have found a correlation between safety culture measured by SAQ and patient harm, this relation has yet to be further explored in primary care [78, 79]. Some studies found associations between patient safety culture and adverse event reports. One study found that more events and near misses were reported when there was a strong culture of safety [54]. This supports the widely held belief that low rates of incidence reporting are linked to poor safety climate and higher levels of patient risk. However, another study found that fewer numbers of medication errors were reported with higher scores of Teamwork climate, Safety climate, Job satisfaction, Perception of management, Working condition and overall safety culture [47]. Only for the dimension Stress recognition, greater numbers of medication errors were reported as Stress recognition increased [47]. Due to few inconsistent results, exploring adverse event rates in primary care and how rates in specific work sites correlate with safety culture measurements is warranted and can also motivate improvement effort.

With the increasing need for care and nursing in an aging population, studies supporting the practical application of patient safety measures in home care and nursing homes are highly welcome in the future. However, the concept of safety climate may be relevant for all organizations that operate with risk [80]. Variance in safety climate is therefore relevant to assess in other public sectors that operate with risk or care for vulnerable users, like for example child protection services [81]. It could also be relevant for social services, the police sector, and services for unemployed and for refugees.

## Conclusion

A systematic review was conducted, and the studies were divided into four analytic themes: 1) validation analysis, 2) descriptive analysis, 3) variance assessment, and 4) intervention evaluation. The synthesis demonstrated that SAQ is valid for use in primary care, but it is important to adapt and validate the questionnaire to the specific setting and participants under investigation. Moreover, differences in SAQ factor scores were related to a variety of descriptive factors, that should be considered in future studies More studies, especially variance and intervention studies, are warranted in primary care.

## Data Availability

All data generated or analysed during this study are included in this published article.

## Abbreviations

CSAQ: Chinese version of safety attitudes questionnaire
CUSP: Comprehensive Unit-based Safety Program
ICC: Intraclass correlation
ICU: Intensive care units
SAQ: Safety attitudes questionnaire
SAQ-A(V): Safety attitudes questionnaire ambulatory version
SAQ-SF: Safety attitudes questionnaire short form
